# DJ-1 Promotes Diabetic Corneal Epithelial Wound Healing by Attenuating Hyperglycemia-Induced Oxidative Stress Through Inhibiting PTEN

**DOI:** 10.1167/iovs.66.9.20

**Published:** 2025-07-08

**Authors:** Haoyu Li, Hanhan Peng, Benteng Ma, Xinyue Sun, Liwei Zhang, Baihua Chen

**Affiliations:** 1 Department of Ophthalmology, The Second Xiangya Hospital, Central South University, Changsha, P. R. China; 2 Hunan Clinical Research Centre of Ophthalmic Disease, Changsha, P. R. China

**Keywords:** diabetic keratopathy, corneal epithelial cells, oxidative stress, mitochondria, DJ-1

## Abstract

**Purpose:**

Diabetic keratopathy (DK) is characterized by delayed corneal epithelial wound healing and impaired nerve regeneration, primarily due to mitochondrial oxidative stress. DJ-1 plays a key role in redox regulation. This study explores the effects of DJ-1 downregulation on DK and its mechanisms.

**Methods:**

Type 1 diabetes was induced in male C57BL/6J mice, and DJ-1 was overexpressed. Corneal oxidative stress and activity were assessed using DHE, Ki67, and TUNEL staining. Epithelial repair and nerve regeneration were evaluated by epithelial wound healing and nerve staining. Human corneal epithelial cells (HCE-T) and primary human corneal epithelial cells were exposed to high-glucose conditions, while DJ-1 and phosphatase and tensin homolog (PTEN) expression were modulated in HCE-T cells. Mitochondrial alterations were assessed by transmission electron microscopy, mitochondrial membrane potential staining, and mitoSOX staining. DJ-1, PTEN, and antioxidant protein levels were measured by immunofluorescence and Western blotting.

**Results:**

In diabetic mice and high glucose–treated cells, DJ-1 and antioxidant enzyme levels were significantly reduced, while PTEN expression increased, accompanied by mitochondrial structural and functional impairments. DJ-1 overexpression alleviated oxidative stress and apoptosis, enhanced cell proliferation, and promoted epithelial wound healing and nerve regeneration. In HCE-T cells, DJ-1 downregulated PTEN, upregulated antioxidant proteins, and restored mitochondrial function, reducing reactive oxygen species accumulation and activity loss caused by high glucose. PTEN activation under high glucose diminished DJ-1’s protective effects. DJ-1 also directly interacted with PTEN, indicating a regulatory mechanism.

**Conclusions:**

DJ-1 deficiency disrupts mitochondrial function, upregulates PTEN, and suppresses antioxidant protein expression, exacerbating corneal oxidative stress. These findings provide insights into molecular mechanisms underlying DK.

Diabetes mellitus (DM) is a chronic metabolic condition characterized by insulin deficiency or resistance, leading to sustained hyperglycemia.^1^ Ocular complications associated with DM significantly diminish patients’ quality of life and can result in vision loss.^2^ Although diabetic retinopathy is the most widely researched ocular complication of DM, other diabetes-related eye disorders often receive less attention. DM adversely impacts the ocular surface, potentially contributing to visual impairment.^3^ Clinically, diabetic patients frequently exhibit symptoms such as diminished corneal sensitivity, superficial punctate keratitis, and delayed corneal epithelial wound healing, collectively referred to as diabetic keratopathy (DK).^4^ DK has been reported in 47% to 64% of patients with DM.^5^ Nevertheless, the precise mechanisms responsible for DK remain poorly elucidated.

The development of DK involves multiple interrelated processes, including oxidative stress, decreased neurotrophic factors, mitochondrial dysfunction, and other molecular pathways.^6^^–^^9^ Hyperglycemia, a hallmark of DM, is closely linked to mitochondrial dysfunction and oxidative stress.^10^^,^^11^ Mitochondria are the main generators of reactive oxygen species (ROS) within cells and are highly vulnerable to damage caused by oxidative stress.^12^^,^^13^ Oxidative stress arises when mitochondrial dysfunction disrupts the equilibrium between ROS production and the antioxidant defense system's ability to neutralize them.^14^ The cornea, due to its high metabolic activity, is particularly prone to ROS-induced damage. Studies have shown that under hyperglycemia conditions, ROS and oxidative stress markers, such as 8-hydroxydeoxyguanosine, are elevated in diabetic corneas, while levels of antioxidant enzymes (e.g., MnSOD and Cu/ZnSOD) are reduced, leading to increased apoptosis of corneal epithelial cells and nerves.^15^^–^^18^ Consequently, identifying targets to counteract oxidative stress is essential for the prevention and treatment of DK.

DJ-1, which is encoded by the *PARK7* gene, was first discovered in individuals with Parkinson's disease, where it is associated with worsening oxidative stress and contributing to the degeneration of dopaminergic neurons.^19^^,^^20^ This protein serves multiple functions, including mitigating oxidative stress, preserving mitochondrial stability, and modulating gene expression.^21^^–^^23^ Additionally, DJ-1 acts as a key negative regulator of phosphatase and tensin homolog (PTEN).^24^^,^^25^ Studies have shown that elevated PTEN expression is linked to damage in corneal nerve fibers, reduced sensitivity, and delays in corneal epithelial and endothelial wound healing.^26^^,^^27^ Our prior research has highlighted DJ-1’s role in alleviating oxidative stress in diabetic retinopathy.^28^^–^^30^ Based on these findings, we hypothesized that PTEN activation, driven by the suppression of DJ-1 expression under high-glucose (HG) conditions, leads to oxidative stress in corneal epithelial cells, thereby leading to DK.

In this study, we sought to explore the effects of DJ-1 on corneal epithelial cells under HG conditions and to uncover the underlying molecular mechanisms through both in vivo and in vitro experiments. Our findings revealed that DJ-1 expression is suppressed in diabetic corneas, accompanied by PTEN activation. Increasing DJ-1 expression significantly improved the activity and mitochondrial function of diabetic corneal epithelial cells and promoted corneal nerve regeneration, whereas PTEN activation inhibited these effects. This study sheds light on the molecular mechanisms by which DJ-1 regulates mitochondrial function and oxidative stress in corneal epithelial cells via PTEN, offering novel insights for the treatment of DK.

## Materials and Methods

### Animals

To avoid the influence of sex hormones, male C57BL/6J mice aged 8 weeks were obtained from Hunan SJA Laboratory Animal Co., Ltd. (Changsha, China). The experimental protocols were reviewed and approved by the Institutional Animal Care and Use Committee and conducted in accordance with the ARVO Statement for the Use of Animals in Ophthalmic and Vision Research. Type 1 diabetes mellitus was induced through intraperitoneal administration of streptozotocin (STZ, 50 mg/kg; Sigma-Aldrich, St. Louis, MO, USA) over 5 consecutive days. Blood glucose levels were monitored, and mice exhibiting levels exceeding 350 mg/dL at 16 weeks post-STZ injection were selected for further studies. To evaluate the protective role of DJ-1, adeno-associated virus was injected subconjunctivally to overexpress DJ-1. Both negative control and overexpression vectors were designed and provided by OBiO Technology (Shanghai, China).

### Induction of Corneal Epithelial Wounds and Sensitivity Assessment

Corneal epithelial wounds were created by removing a 2.5-mm diameter area of the central corneal epithelium from anesthetized normal and diabetic mice using an Algerbrush II (Katena Products, Lago Vista, TX, USA). The extent of epithelial defects was monitored at 0, 24, and 48 hours using 0.25% fluorescein sodium, and the defect area was measured with ImageJ software (National Institutes of Health, Bethesda, MD, USA). Corneal sensitivity was evaluated using a Cochet-Bonnet esthesiometer (Luneau Ophtalmologie, Chartres Cedex, France) in conscious mice 7 days postinjury. The sensitivity threshold was defined as the maximum filament length eliciting a positive response.

### Whole-Mount Corneal Staining

To examine nerve regeneration, whole-mount corneal staining was conducted 7 days after wounding. Mouse eyeballs were fixed in Zamboni solution for 2 hours, followed by separation of the cornea. The corneas were blocked and then incubated with an anti-β III Tubulin antibody (1:500; CST, Danvers, MA, USA) overnight at 4°C. Subsequently, the corneas were treated with DyLight 488–conjugated secondary antibody (1:500; Boster, Wuhan, China) for 1 hour at room temperature. After mounting, the corneas were imaged using a fluorescence microscope (Axio Imager M2; Carl Zeiss, Jena, Germany), and nerve fiber density was quantified using ImageJ software.

### Detection of Oxidative Stress via Dihydroethidium Staining

Oxidative stress in the cornea was evaluated using dihydroethidium (DHE) staining according to the instructions. Frozen eyeball sections were incubated with 1 µM DHE (Biosharp, Beijing, China) at 37°C for 30 minutes and visualized under a fluorescence microscope.

### TUNEL Assay for Apoptosis Detection

Apoptosis was analyzed using a TUNEL detection kit (Vazyme, Nanjing, China). Briefly, eyeballs were collected and sectioned to prepare frozen samples. The sections were fixed with 4% paraformaldehyde, permeabilized using PBS containing 0.1% Triton X-100, and then incubated with TdT reaction mix for 1 hour in a dark, humidified environment at 37°C. Following counterstaining with DAPI (Genview, Shanghai, China), the sections were imaged under a fluorescence microscope, and the percentage of TUNEL-positive cells was quantified.

### Immunofluorescence Staining

Immunofluorescence staining was performed using specific rabbit anti-DJ-1 (1:200; Selleck, Shanghai, China), anti-PTEN (1:200; PTMbio, Hangzhou, China), and anti-Ki67 (1:200; HuaBio, Hangzhou, China) primary antibodies. Eyeballs were embedded in OCT medium and sectioned. The resulting frozen sections were fixed in 4% paraformaldehyde for 15 minutes. After washing with PBS, blocking and permeabilization were performed. The sections were incubated with primary antibodies overnight at 4°C, followed by treatment with DyLight-conjugated secondary antibodies (1:500; Boster, Wuhan, China). After counterstaining with DAPI, the samples were visualized using a fluorescence microscope.

### Cell Culture and Treatment

In this study, we utilized immortalized human corneal epithelial cells (HCE-T) and primary human corneal epithelial cells (HCECs). The HCE-T cell line (CTCC-002-0014; Zhejiang Meisen Cell Technology Co., Ltd., Jinhua, China) was cultured in Dulbecco's modified Eagle's medium (BasalMedia, Shanghai, China) supplemented with 10% fetal bovine serum (FBS) and 1% antibiotic-antimycotic from Gibco (Waltham, MA, USA) at 37°C with 5% CO_2_. Primary HCECs were isolated from discarded corneoscleral specimens obtained from the eye bank of the Second Xiangya Hospital, Central South University, following established protocols.^31^^,^^32^ Both HCE-T cells and primary HCECs were exposed to either HG (30 mM D-glucose) or normal glucose (NG, 5.6 mM D-glucose) conditions for 48 hours after starvation. In the case of HCE-T cells, 30 mM mannitol was used as an osmotic control. For primary HCECs, the osmolarity of the NG medium was adjusted to match that of the HG medium by adding mannitol. HCE-T cells were treated with sodium phenylbutyrate (150 µM) to modulate DJ-1 activity and oroxin B (2 µM) to regulate PTEN signaling.

### Protein Extraction and Western Blotting

Proteins were extracted from HCE-T cells and corneal epithelium using RIPA lysis buffer (Servicebio, Wuhan, China) containing PMSF (Solarbio, Beijing, China). Proteins were separated using SDS-PAGE and subsequently transferred onto PVDF membranes (Millipore Sigma, St. Louis, MO, USA). The membrane was then washed and blocked with 5% skimmed milk. Primary antibodies against DJ-1 (1:2000; Selleck), PTEN (1:2000; PTMBio), GCLC (1:1000; Abcam, Cambridge, MA, USA), MnSOD (1:1000; Selleck), catalase (1:1000; CST), and β-actin (1:5000; Sigma-Aldrich) were used. The bound primary antibody was detected by horseradish peroxidase–conjugated secondary antibodies and the ChemiDoc MP System (Bio-Rad, Hercules, CA, USA). Protein bands were quantified using ImageJ software.

### Mitochondrial Membrane Potential Assay

As described previously, mitochondrial membrane potential (*ΔΨm*) was measured using the JC-1 detection kit (Biosharp, Beijing, China).^33^ HCE-T cells were incubated with JC-1 for 30 minutes at 37°C. After being washed three times with an assay buffer, cells were then analyzed using a fluorescence microscope (Leica, Wetzlar, Germany). JC-1 monomer and dimer fluorescence intensity were measured using ImageJ software.

### Mitochondrial ROS Staining

Mitochondrial ROS were detected using the MitoSOX Red staining kit (MedChemExpress, Shanghai, China). PBS-washed cells were incubated with 5 µM MitoSOX for 30 minutes at room temperature and counterstained with Hoechst 33342 (Beyotime, Shanghai, China). Fluorescence intensity was measured using ImageJ software.

### Transmission Electron Microscopy

Mitochondrial structure was analyzed using transmission electron microscopy (TEM). Cells were first fixed in 2.5% glutaraldehyde, followed by dehydration using an ethanol gradient and subsequent embedding. Ultrathin sections were analyzed using a transmission electron microscope (Hitachi, Tokyo, Japan) operating at 80 kV. Mitochondrial dimensions were quantified using ImageJ software.

### Cell Migration Analysis

The cell migration ability was evaluated by the wound-healing assay. As an adherent confluent monolayer formed, a uniform wound was created using a 200-µL pipette tip. Cells were maintained in FBS-free experimental medium, and wound closure was monitored using a light microscope. Migration was quantified using ImageJ software.

### Protein Interaction Analysis

The protein sequence information for DJ-1 and PTEN in humans and mice was obtained from UniProt (https://www.uniprot.org/). Structural modeling was performed using the AlphaFold Server,^34^ and visualization was conducted using PyMOL Molecular Graphics System, Version 3.0.4 (Schrödinger, LLC, New York, NY, USA). Co-immunoprecipitation (Co-IP) was employed to confirm the interaction between DJ-1 and PTEN. In brief, cells were lysed using immunoprecipitation cell lysis buffer (New Cell & Molecular Biotechnology, Suzhou, China). Ten percent of the lysate was reserved as input, and IgG (Beyotime) served as a negative control. Immunocomplexes were incubated with protein A/G magnetic beads (Beyotime) and eluted by boiling in loading buffer at 100°C for 5 minutes. Protein interactions were then analyzed by Western blotting.

### Statistical Analysis

Data analysis was performed using GraphPad Prism (v9.0.1; GraphPad Software, La Jolla, CA, USA), with results presented as mean ± SD. Statistical significance was assessed using a two-tailed unpaired Student's *t*-test or one-way ANOVA followed by Tukey's multiple comparisons test. A *P* value of less than 0.05 was considered statistically significant.

## Results

### Hyperglycemia Disrupted DJ-1, PTEN, and Antioxidant Enzyme Expression in the Corneal Epithelium

**Figure 1.**
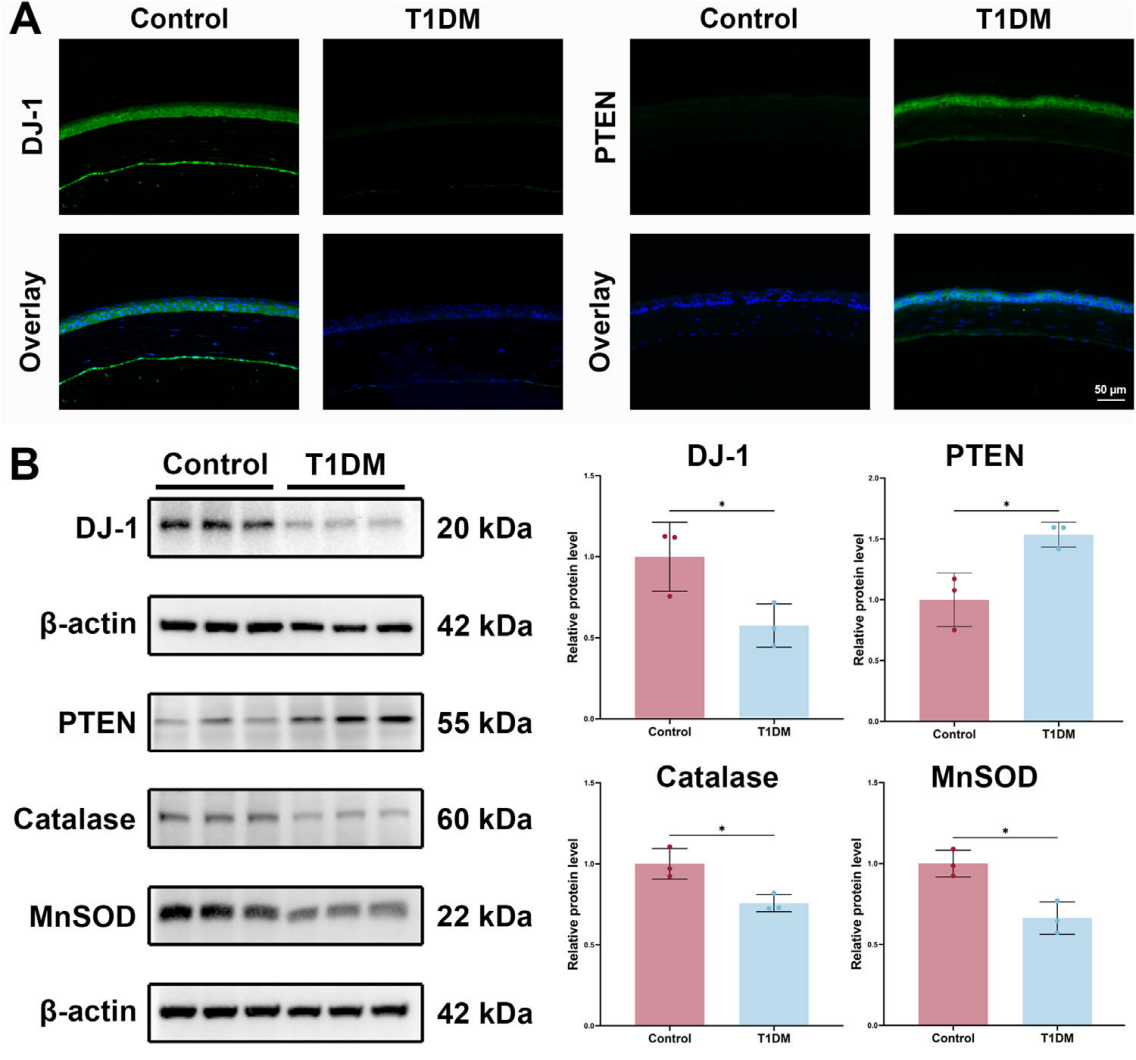
Hyperglycemia disrupts DJ-1, PTEN, and antioxidant enzymes expression in the corneal epithelium. (**A**) Immunofluorescence staining was performed to evaluate the distribution of DJ-1 and PTEN in the corneas of diabetic and control mice. *Scale bars*: 50 µm. (**B**) Western blotting analysis of DJ-1, PTEN, catalase, and MnSOD protein levels in corneas of diabetic and control mice (*n* = 3). *P < 0.05. T1DM, type 1 diabetes mellitus.

To assess the expression of DJ-1, PTEN, and antioxidant enzymes, corneal epithelial tissues from wounded corneas of mice were collected and subjected to immunofluorescence staining and western blotting (WB). Immunofluorescence staining demonstrated that both DJ-1 and PTEN proteins were present in the cornea, particularly within the corneal epithelium. The body weight and blood glucose levels of mice in both the control and diabetic groups, 16 weeks after modeling, are shown in Supplementary Figure S1. The results indicated a marked reduction in DJ-1 expression and a significant increase in PTEN expression in diabetic corneas (Fig. 1A). Additionally, the dysregulation of DJ-1 and PTEN was associated with a substantial decrease in the expression of antioxidant enzymes, catalase and MnSOD, in the diabetic cornea (Fig. 1B). These observations suggest that DJ-1 and antioxidant protein expression were downregulated, while PTEN expression was elevated, pointing to an imbalance in redox homeostasis during diabetic corneal epithelial repair.

### DJ-1 Enhanced the Repair of Corneal Epithelial Wounds and Facilitated Nerve Regeneration in Diabetic Mice

**Figure 2.**
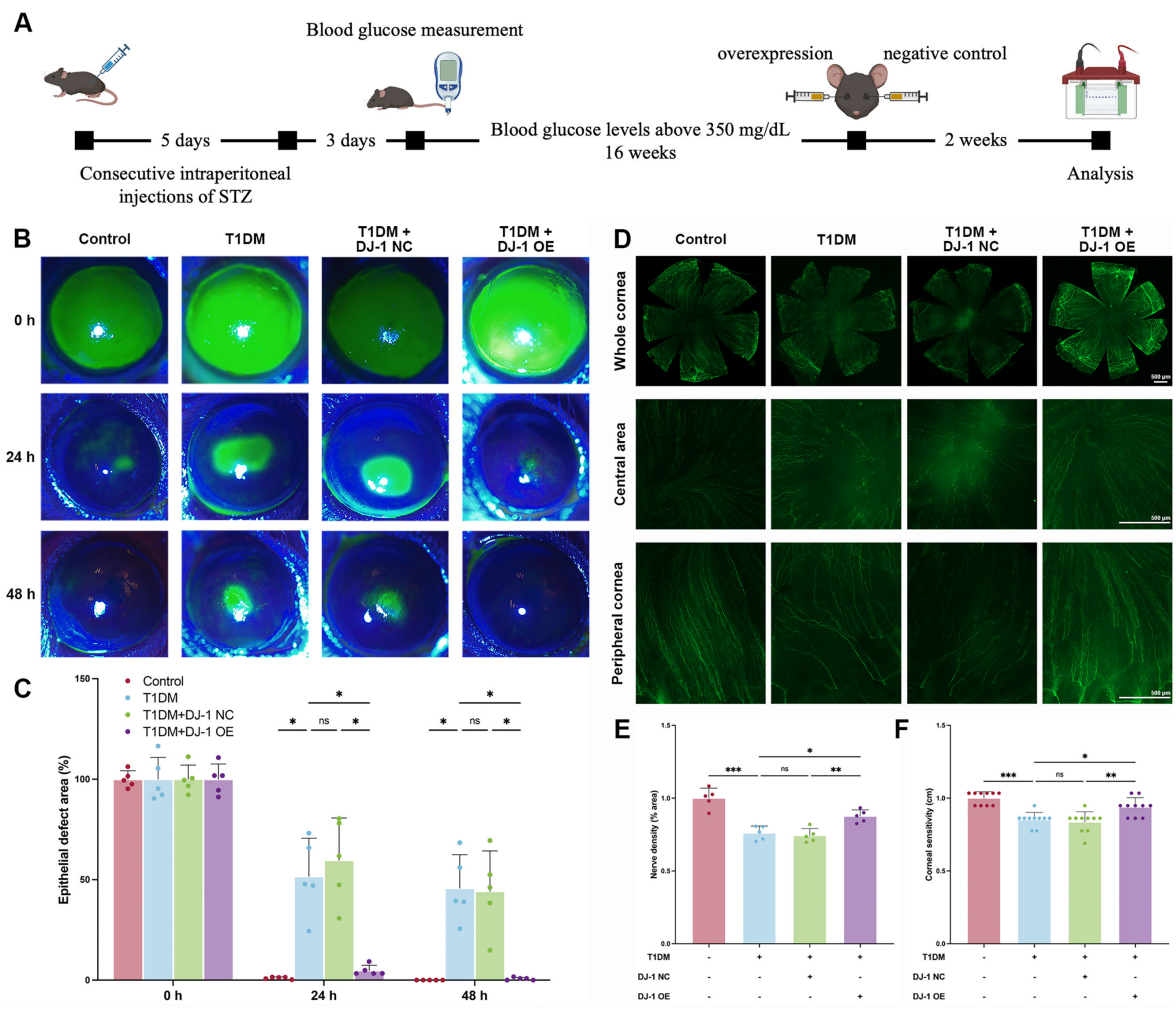
DJ-1 promotes corneal epithelial wound healing and nerve regeneration in diabetic mice. (**A**) Schematic representation of the experimental design. (**B**) Corneal epithelial wound-healing rates were assessed in control, diabetic, diabetic with control virus, and diabetic with DJ-1 overexpression groups (*n* = 5). (**C**) Quantification of epithelial wound-healing rates. (**D**) Corneal nerve density was analyzed using flat-mount staining across experimental groups (*n* = 5). *Scale bars*: 500 µm. (**E**) Quantitative analysis of corneal nerve density. (**F**) Corneal sensitivity was measured in experimental groups (*n* = 10). *P < 0.05, **P < 0.01, ***P < 0.001; ns, not significant. NC, negative control; OE, overexpression.

During the healing process of diabetic corneal epithelium, the expression of antioxidant proteins, such as DJ-1, is diminished, potentially contributing to the delayed wound repair observed in DK. To investigate the role of DJ-1 in facilitating corneal epithelial repair in diabetic mice, we overexpressed DJ-1 on the ocular surface. The animal experiment process is shown in Figure 2A. The findings revealed that diabetic corneal epithelial healing was significantly slower compared to the control group (Figs. 2B, 2C). In contrast, overexpression of DJ-1 markedly accelerated the healing process in diabetic corneal epithelium, whereas the control vector had no significant effect (Figs. 2B, 2C).

In addition to impairing epithelial repair, hyperglycemia significantly inhibits corneal nerve regeneration. The whole-mount staining results indicated that diabetic mice had significantly reduced corneal nerve density and abnormal central corneal nerve distribution compared to the control group (Figs. 2D, 2E). Overexpression of DJ-1 significantly promoted nerve regeneration in diabetic corneas, while the control vector showed no notable effect (Figs. 2D, 2E). Consistent with nerve regeneration, DJ-1 overexpression facilitated the recovery of central corneal sensitivity in diabetic mice (Fig. 2F).

### DJ-1 Alleviated Oxidative Stress in Diabetic Corneas

**Figure 3.**
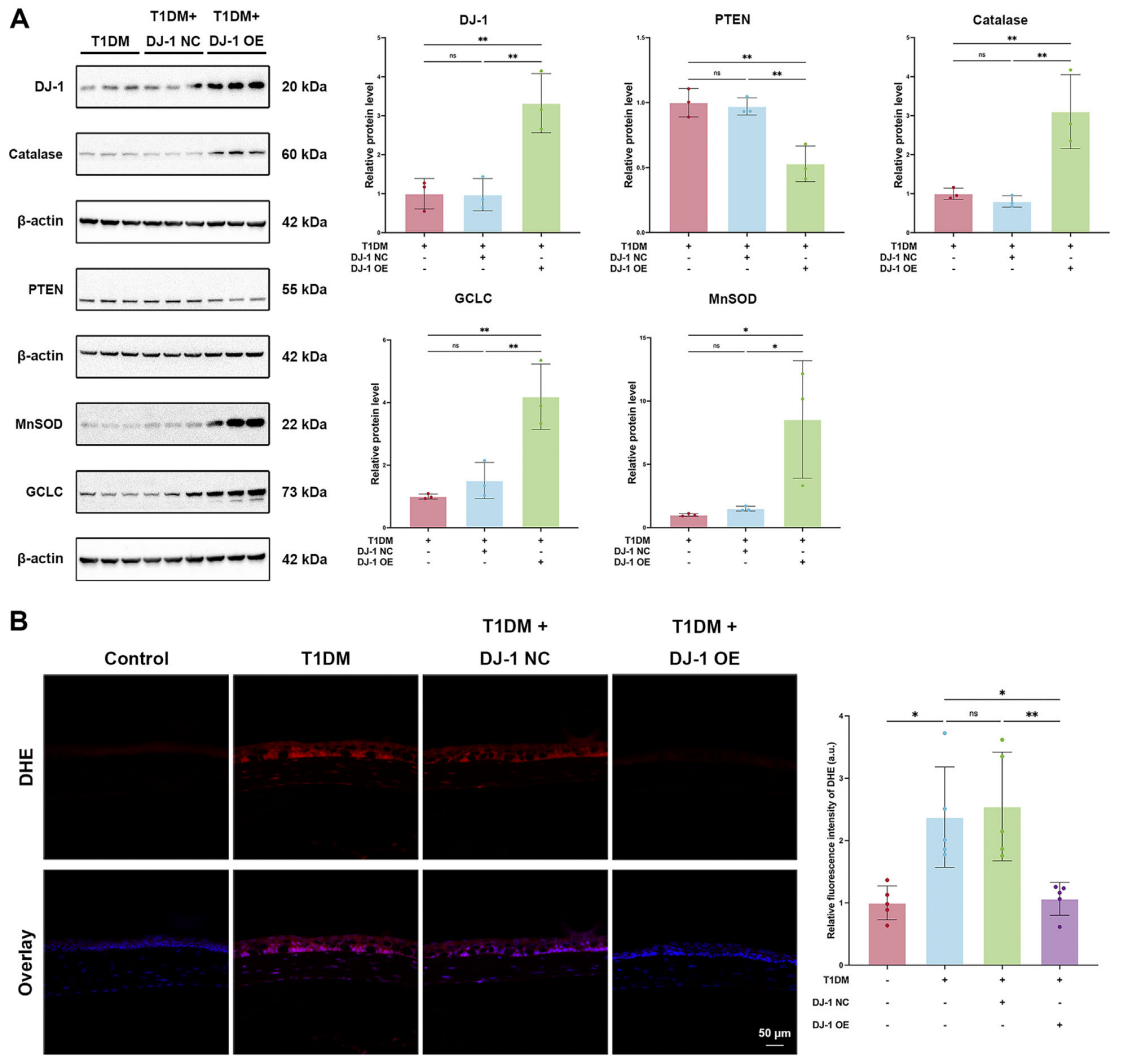
DJ-1 attenuates oxidative stress in diabetic corneas. Oxidative stress was assessed using (**A**) Western blotting (*n* = 3) and (**B**) DHE staining (*n* = 5), respectively, in control, diabetic, diabetic with control virus, and diabetic with DJ-1 overexpression groups. *Scale bars*: 50 µm. *P < 0.05, **P < 0.01; ns, not significant.

Mitochondrial dysfunction plays a critical role in the accumulation of ROS, oxidative stress, and apoptosis. As a mitochondrial protein, DJ-1 is closely associated with redox balance and apoptosis. To further investigate the mechanisms by which DJ-1 enhances corneal epithelial repair and nerve regeneration, we examined its effects on oxidative stress in diabetic corneal epithelial cells. As previously mentioned, the expression of antioxidant proteins in the corneal epithelium of diabetic mice was significantly lower than in the control group. However, localized overexpression of DJ-1 effectively restored the levels of antioxidant enzymes, including catalase, MnSOD, and GCLC, while reducing PTEN expression in the corneal epithelium (Fig. 3A). Concurrently, the DHE expression in the diabetic corneas was significantly increased, indicating increased oxidative stress (Fig. 3B). As expected, DJ-1 overexpression significantly reduced DHE levels in diabetic corneas, while the control vector had no such effect (Fig. 3B).

### DJ-1 Promoted Proliferation and Alleviated Apoptosis in Diabetic Corneas

**Figure 4.**
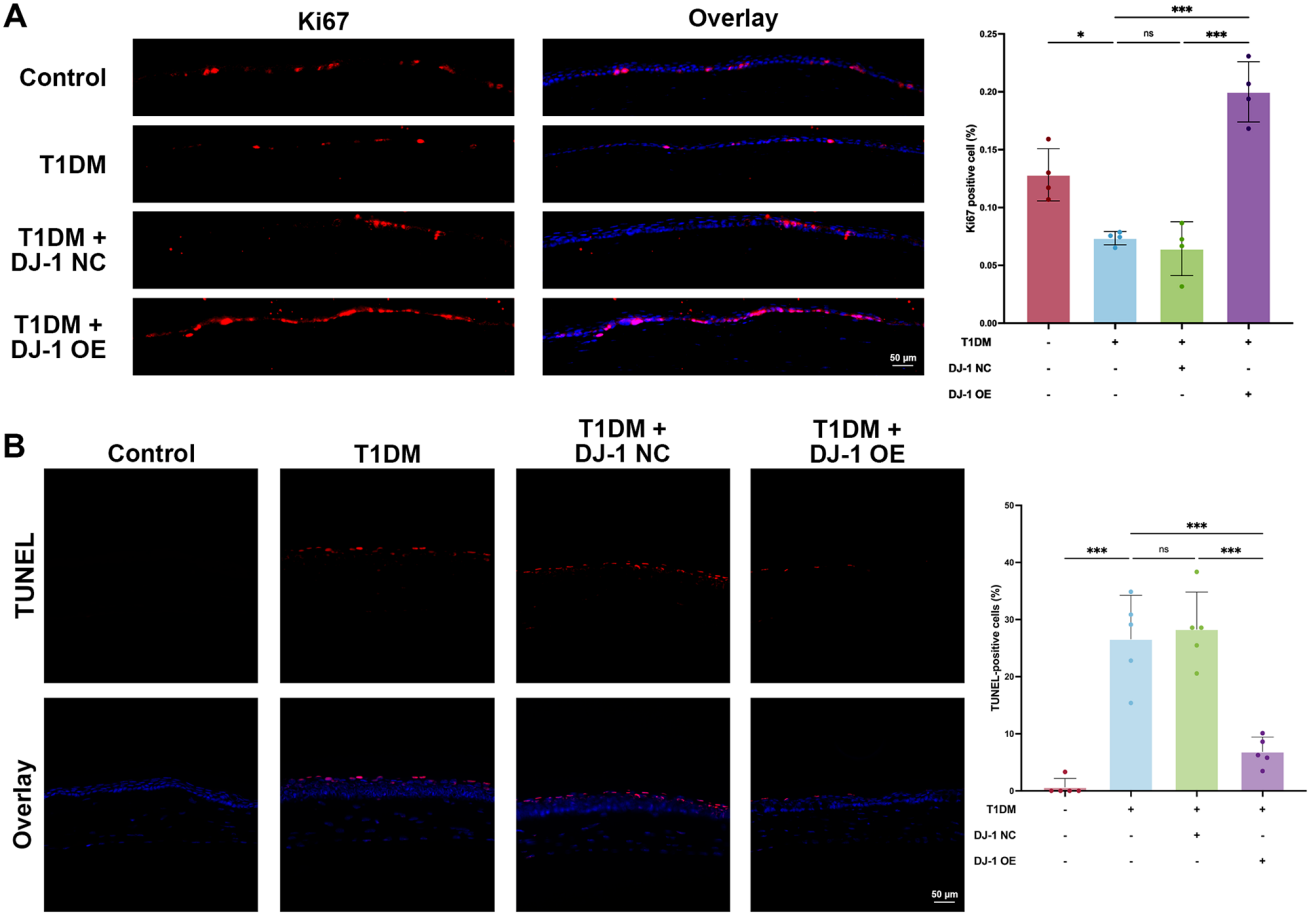
DJ-1 promotes proliferation and alleviates apoptosis in diabetic corneas. (**A**) Ki67 immunofluorescence staining was used to evaluate corneal epithelial cell proliferation in control, diabetic, diabetic with control virus, and diabetic with DJ-1 overexpression groups (*n* = 4). *Scale bars*: 50 µm. (**B**) TUNEL staining was used to evaluate corneal epithelial cell apoptosis in control, diabetic, diabetic with control virus, and diabetic with DJ-1 overexpression groups (*n* = 5). *Scale bars*: 50 µm. *P < 0.05, ***P < 0.001; ns, not significant.

Consistent with previous findings, immunofluorescent staining for the proliferation marker Ki67 revealed that the proliferative activity of diabetic corneal epithelial cells was reduced.^35^ DJ-1 overexpression notably elevated the number of Ki67-positive cells in the diabetic corneal epithelium, indicating enhanced corneal epithelial cell proliferation (Fig. 4A). Additionally, the number of TUNEL-positive apoptotic cells in diabetic corneas was significantly higher, with apoptosis primarily localized in the epithelium—particularly in superficial cells—as well as in the stroma and endothelium (Fig. 4B). DJ-1 overexpression significantly reduced TUNEL-positive cells in diabetic corneas, suggesting its antiapoptotic ability (Fig. 4B).

### DJ-1 Enhanced HCE-T Cell Migration Under HG Conditions by Regulating PTEN

**Figure 5.**
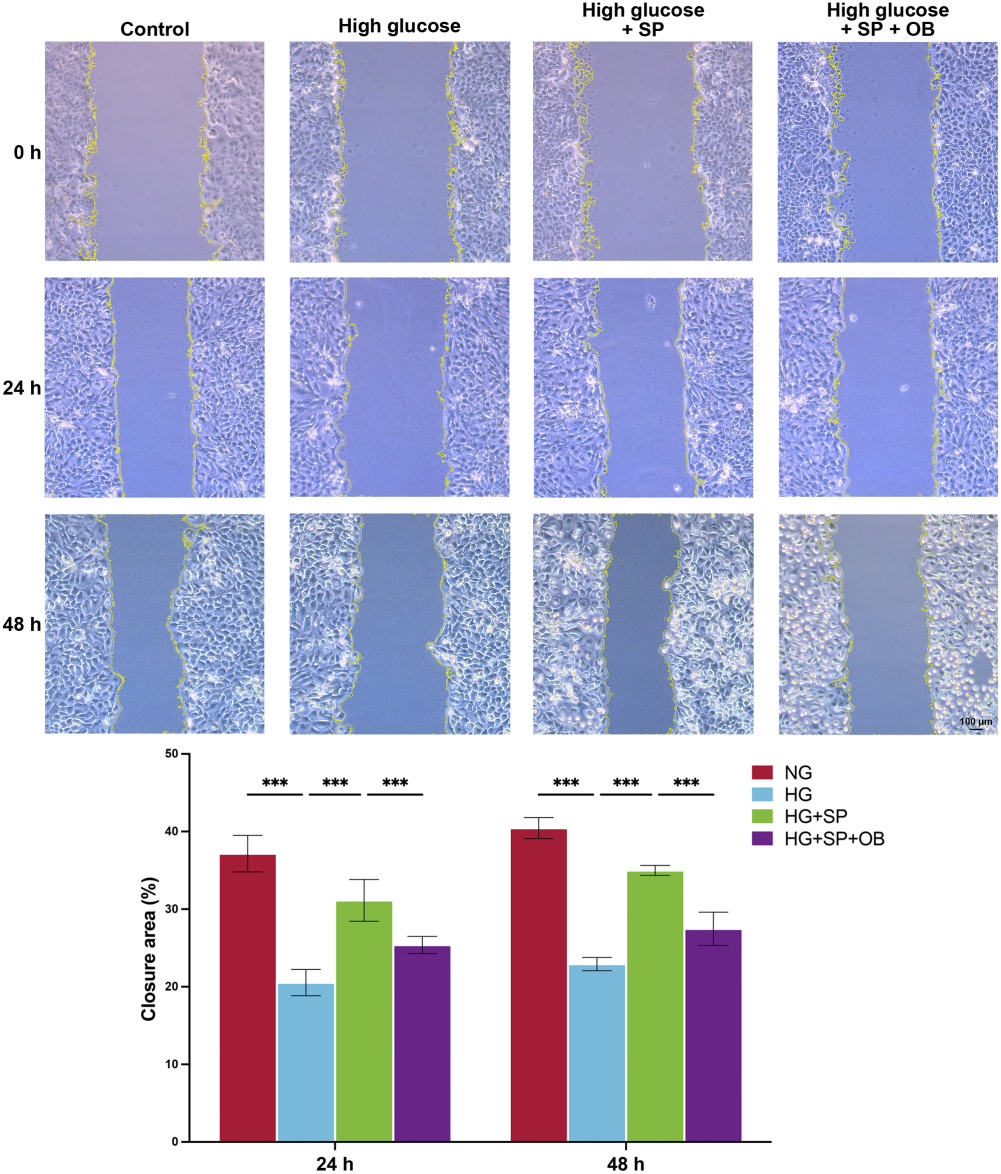
DJ-1 enhances HCE-T cell migration under HG conditions by regulating PTEN. A cell scratch assay was conducted to evaluate cell migration under HG, mannitol (osmotic pressure control), and normal conditions (*n* = 4). *Scale bars*: 100 µm. ***P < 0.001. OB, oroxin B; SP, sodium phenylbutyrate.

The repair of corneal epithelial wounds is predominantly driven by the migration of corneal epithelial cells. Consistent with in vivo findings, HG significantly suppressed the migration of HCE-T cells (Fig. 5). Overexpression of DJ-1 mitigated this inhibitory effect. However, when PTEN expression was upregulated, the protective effect of DJ-1 on cell migration was negated.

### DJ-1 Alleviated HG-Induced Mitochondrial Dysfunction in HCE-T Cells by Regulating PTEN

**Figure 6.**
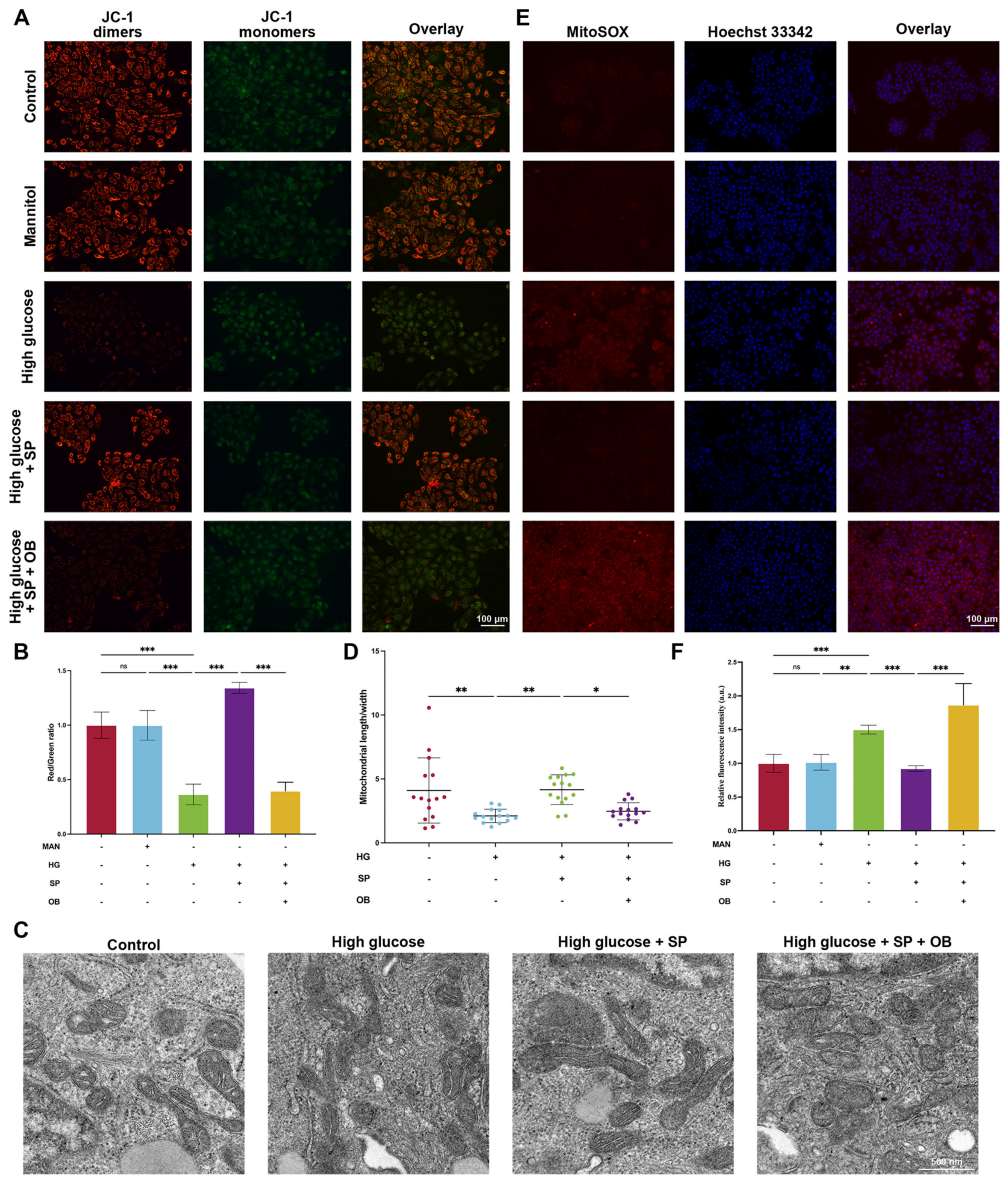
DJ-1 alleviates HG-induced mitochondrial dysfunction in HCE-T cells by regulating PTEN. (**A**) JC-1 staining was used to detect the mitochondrial membrane potential of HCE-T cells under different conditions (*n* = 6). *Scale bars*: 100 µm. (**B**) Quantification of JC-1 dimer-to-monomer ratio. (**C**) Electron microscopy images depicting mitochondrial morphology under various conditions. *Scale bars*: 500 nm. (**D**) Statistical analysis of mitochondrial length-to-width ratio (*n* = 15). (**E**) MitoSOX staining was used to measure mitochondrial reactive oxygen species levels (*n* = 5), with fluorescence intensity quantification shown in **F**. *Scale bars*: 100 µm. *P < 0.05, **P < 0.01, ***P < 0.001; ns, not significant.

To further explore the impact of DJ-1 on corneal epithelial wound healing under HG, JC-1 staining was used to assess mitochondrial function. In apoptotic or necrotic cells, the *ΔΨm* decreases, causing JC-1 to diffuse from the mitochondria and produce monomers, which result in a reduced red/green fluorescence ratio. As indicated, HG significantly decreased the JC-1 red/green ratio and mitochondrial length-to-width ratio compared to the control group (Figs. 6A–D). However, upregulating DJ-1 expression alleviated this HG-induced damage in *ΔΨm* and morphology. As expected, increasing PTEN expression levels counteracted the protective effect of DJ-1. Consistently, HG induced mitochondrial shrinkage, reduced and thickened inner cristae, and increased membrane electron density in corneal epithelial cells (Fig. 6C). The use of PTEN agonists attenuated the protective effects on mitochondrial structure associated with DJ-1 upregulation. Mitochondria are critical sites of ROS generation. Under HG conditions, mitochondrial function is compromised, leading to elevated ROS levels. To assess the impact of various interventions on mitochondrial ROS, MitoSOX staining was conducted. As shown, HG treatment significantly increased mitochondrial ROS accumulation compared to the control group (Figs. 6E, 6F). However, enhancing DJ-1 expression effectively reduced mitochondrial ROS levels. Conversely, when PTEN expression was stimulated alongside increased DJ-1, mitochondrial ROS accumulation was again promoted.

### DJ-1 Restored Redox Balance in HCECs by Regulating the Antioxidant Protein Profile via PTEN

**Figure 7.**
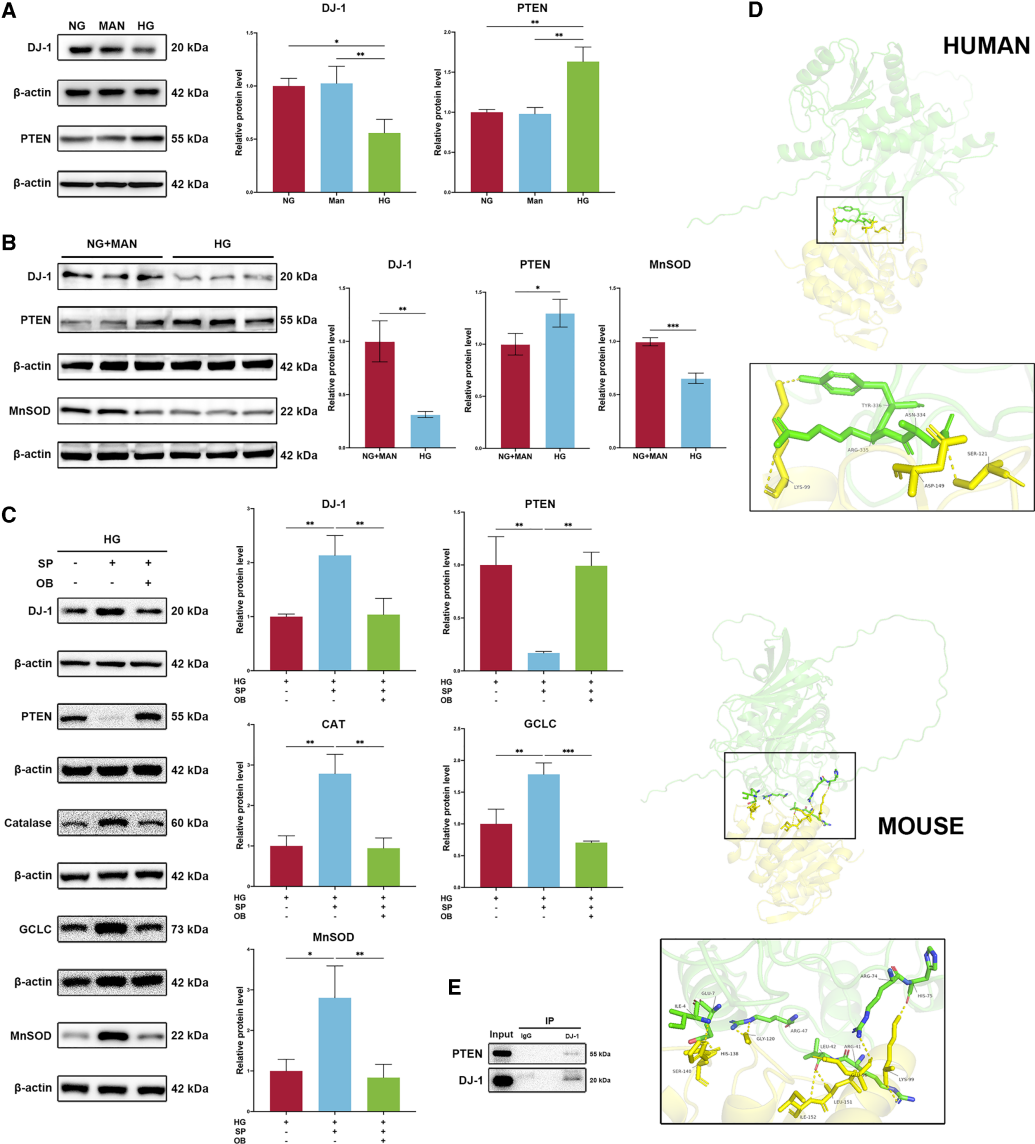
DJ-1 interacts with PTEN protein and alleviates PTEN-induced oxidative stress in corneal epithelial cells under HG conditions. (**A**) Western blotting analysis of DJ-1 and PTEN protein levels under HG, mannitol (osmotic control), and normal conditions in HCE-T cells (*n* = 3). (**B**) Western blotting analysis of DJ-1 and PTEN protein levels under HG and normal (with mannitol) conditions in primary human corneal epithelial cells (*n* = 3). (**C**) Effects of DJ-1 and/or PTEN modulation on the expression of DJ-1, PTEN, catalase, GCLC, and MnSOD under HG conditions (*n* = 3). (**D**) DJ-1 and PTEN protein interaction was predicted using the AlphaFold server for human and mouse proteins, with visualization via PyMOL software. (**E**) Immunoprecipitation was conducted to confirm the DJ-1/PTEN interaction in HCE-T cells. *P < 0.05, **P < 0.01, ***P < 0.001. IP, immunoprecipitation; MAN, mannitol.

Mitochondrial dysfunction and dysregulated antioxidant protein expression under HG conditions contribute to redox imbalance, leading to ROS accumulation and oxidative stress. To explore the roles of DJ-1 and PTEN in regulating antioxidant protein expression under HG conditions, we examined their effects on cellular antioxidant profiles. Consistent with previous studies, mannitol, employed as an osmotic control, did not significantly alter protein expression, with DJ-1 and PTEN levels comparable to the control group. In contrast, HG conditions significantly disrupted the expression of DJ-1 and PTEN in HCE-T cells and primary HCECs (Figs. 7A, 7B). Overexpression of DJ-1 under HG conditions markedly suppressed PTEN expression while significantly enhancing the levels of antioxidant proteins, including catalase, MnSOD, and GCLC (Fig. 7C). However, co-overexpression of DJ-1 and PTEN restored PTEN levels but suppressed DJ-1 and antioxidant protein expression (Fig. 7C). These findings imply that PTEN may regulate DJ-1 expression through feedback mechanisms or potentially interact with DJ-1.

### DJ-1 Interacted With PTEN

DJ-1/PTEN protein–protein complexes of human and mouse species were modeled by AlphaFold, and the best-confidence models were visualized by PyMOL software (Fig. 7D). The prediction results suggest that DJ-1 and PTEN have potential binding ability in both humans and mice. To further verify the direct binding effect between the proteins, we performed a Co-IP experiment. The experimental results showed that in HCE-T cells, DJ-1 protein can bind to PTEN, while IgG does not have this ability (Fig. 7E).

## Discussion

Oxidative stress is a central pathological feature in various diabetic eye complications, including diabetic retinopathy and DK.^16^^,^^33^^,^^36^^,^^37^ Mitochondrial dysfunction, which disrupts redox balance by promoting the accumulation of ROS, is a major contributor to oxidative stress.^38^^–^^41^ Since corneal epithelial cells are directly exposed to the external environment and exhibit high proliferative activity, they are particularly vulnerable to oxidative damage.^39^^,^^42^ In this study, we examined mitochondrial damage and oxidative stress in corneal epithelial cells associated with DK at both animal and cellular levels. The results demonstrated a reduction in mitochondrial antioxidant proteins, including DJ-1, catalase, and MnSOD, along with an increase in mitochondrial ROS under HG conditions. In contrast, PTEN expression was elevated, suggesting that the DJ-1/PTEN signaling pathway plays a crucial role in regulating antioxidant protein expression during oxidative stress in DK.

Under normal conditions, redox balance is maintained by antioxidant proteins and ROS scavenging mechanisms. DJ-1, a mitochondrial protein, is essential for redox regulation and cellular signaling.^28^^,^^29^^,^^33^^,^^43^ Previous studies have demonstrated that DJ-1/Nrf2 signaling is involved in the corneal endothelium, particularly in type 2 diabetic mice, where DJ-1 downregulation increases the susceptibility of corneal endothelial cells to oxidative damage induced by ultraviolet A.^44^^–^^46^ However, the impact of DJ-1 downregulation on the corneal epithelium remains unclear. In our study, we found that under HG conditions, both DHE levels and apoptosis were significantly increased in mouse corneal epithelial cells. Furthermore, DJ-1 and antioxidant proteins catalase and MnSOD were notably downregulated, indicating that corneal epithelial cells undergo oxidative stress under HG conditions. DJ-1 performs various functions, including acting as a chaperone, protease, transcriptional regulator, redox sensor, mitochondrial homeostasis regulator, and cell signaling modulator.^43^ Previous studies have indicated that DJ-1 overexpression has protective effects.^33^^,^^47^^,^^48^ In this study, we further explored the effects and mechanisms of DJ-1 overexpression in DK. As expected, overexpression of DJ-1 on the ocular surface or in cultured HCE-T cells enhanced antioxidant protein expression and restored mitochondrial membrane potential, improving the activity of corneal epithelial cells. Notably, Nakamura et al.^49^ reported that extracellular DJ-1 can function as a damage-associated molecular pattern, triggering the production of inflammatory cytokines in immune cells. This emphasizes the importance of considering DJ-1 localization and its mechanisms in future studies.

Interestingly, as the expression of DJ-1 changed, PTEN levels showed an opposite trend, suggesting that the effects of DJ-1 may be partly mediated through PTEN regulation. PTEN has been implicated in corneal epithelial and endothelial proliferation. Zhang et al.^27^ reported that intracameral injection of PTEN inhibitors promoted the healing of rat corneal endothelial cell injury, suggesting that the higher PTEN expression in human corneal endothelial cells compared to rats may explain the lower proliferation ability of human cells. Another study showed that miR-23a-3p could inhibit ferroptosis by suppressing PTEN in corneal endothelial cells, providing a potential therapeutic role in Fuchs endothelial corneal dystrophy.^50^ Similarly, inhibition of PTEN promotes corneal epithelial proliferation. For instance, PTEN expression decreases at wound edges during corneal epithelial injury, activating the PI3K/AKT pathway and accelerating repair.^51^ Li et al.^26^ further demonstrated that elevated PTEN expression impairs corneal epithelial regeneration and Akt activation in diabetic mice. In vitro, PTEN inhibition improved corneal epithelial cell migration but not proliferation. Additionally, the PTEN/PI3K/AKT pathway has been linked to the anti-inflammatory effects of quercetin in corneal epithelial cells.^52^ Our findings further confirmed the effect of PTEN on corneal epithelial cells. Overexpression of PTEN greatly weakened the protective effect of DJ-1 overexpressed on HG-induced oxidative stress in corneal epithelial cells. Given that our experimental results suggest DJ-1 may regulate PTEN, we further investigated their interaction. Consistent with previous studies, we confirmed that DJ-1 binds to PTEN in corneal epithelial cells.^25^^,^^53^^,^^54^ DJ-1 appears to inhibit PTEN by directly binding to it and promoting its degradation. Conversely, overactivation of PTEN may lead to the depletion of DJ-1 protein, which could explain our experimental observations.

Peripheral neuropathy affects over half of diabetic patients, with a notable reduction in corneal nerve fiber density and length.^6^^,^^55^^,^^56^ These corneal nerves, which arise from the trigeminal nerve, create a subbasal plexus and extend as free nerve endings into the corneal epithelium.^57^ Importantly, corneal nerve regeneration is markedly slower in diabetic animals during epithelial wound healing,^58^ elevating the risk of anterior segment disorders and infections.^59^ Given the critical role of corneal nerves in maintaining epithelial function, we also investigated the impact of HG-induced oxidative stress on corneal nerves. Our results demonstrated that overexpression of DJ-1 on the ocular surface significantly promoted corneal nerve regeneration and restored corneal sensitivity, which may be a key mechanism for accelerating corneal epithelial wound repair.

The authors acknowledge certain limitations in this study. First, TEM was not employed to observe mitochondria in mouse corneal epithelial cells at the tissue level; instead, observations were confined to HCE-T cells cultured in vitro. Additionally, the study focused exclusively on corneal epithelial cells, without exploring the mechanisms of HG on corneal nerves. The specific mechanisms by which the DJ-1/PTEN signaling pathway affects corneal nerves under HG conditions remain to be elucidated. Due to the limited proliferative capacity of primary HCECs, this study investigated the expression changes of DJ-1, PTEN, and antioxidant enzymes under different conditions in both primary HCECs and HCE-T cells, with functional validation conducted in HCE-T cells. However, the study did not extend these validations to mouse-derived cells. Despite these limitations, the study enhances understanding of corneal epithelial oxidative stress in DK and offers new insights into DJ-1/PTEN signaling as a potential molecular mechanism.

**Figure 8.**
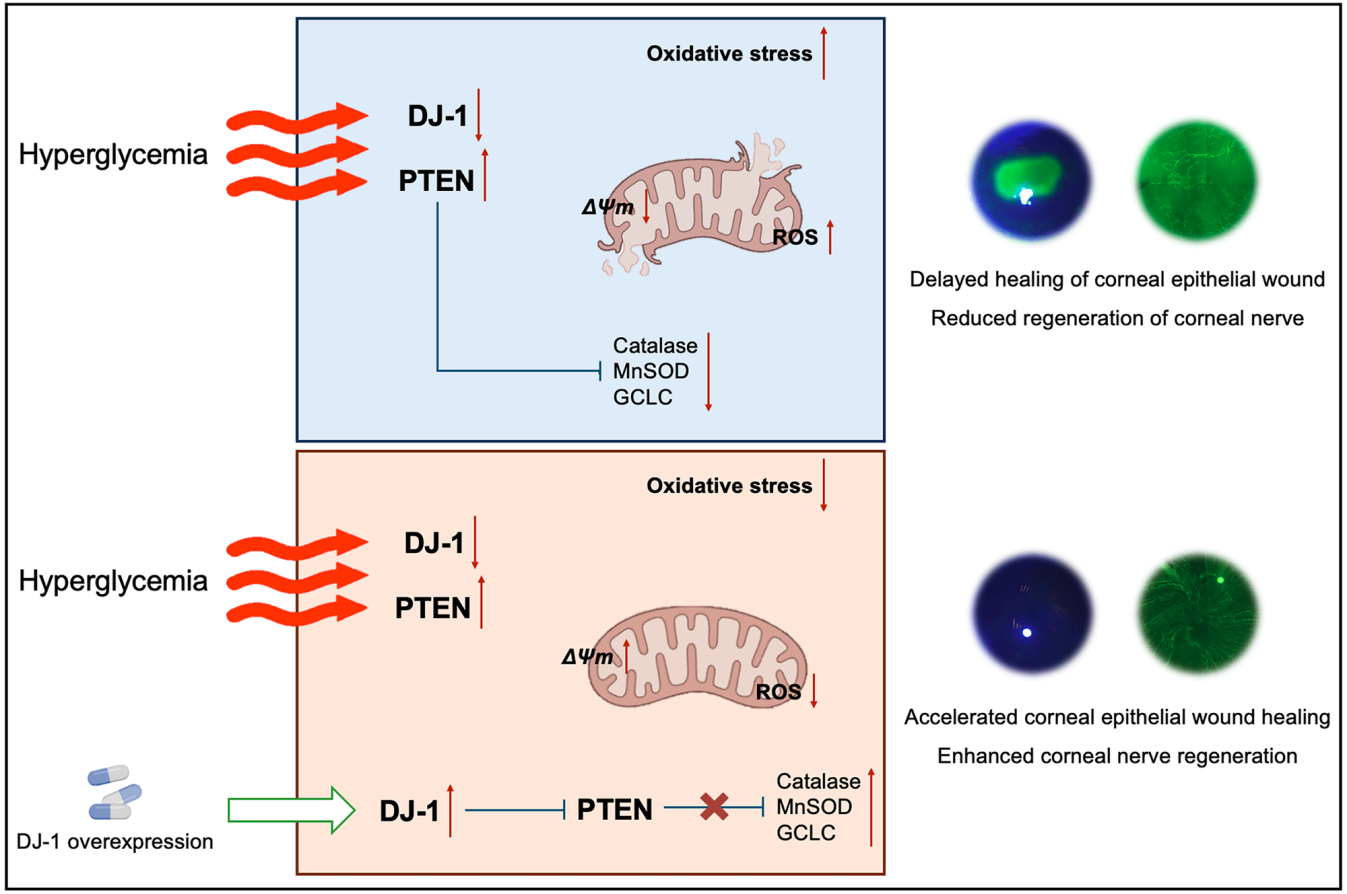
Proposed mechanism by which DJ-1 interacts with PTEN to mitigate PTEN-induced oxidative stress, thereby reducing ocular surface damage in diabetic keratopathy. Chronic hyperglycemia downregulates DJ-1 expression in corneal epithelial cells, resulting in mitochondrial dysfunction, reduced antioxidant protein expression and mitochondrial membrane potential, and increased PTEN levels. This cascade leads to ROS accumulation, oxidative stress, delayed corneal epithelial wound healing, and impaired nerve regeneration, ultimately contributing to diabetic keratopathy.

In summary, our study demonstrates that reduced DJ-1 expression plays a significant role in the oxidative stress associated with DK. Specifically, under HG conditions, decreased DJ-1 levels lead to PTEN overactivation, suppression of antioxidant protein expression, mitochondrial ROS accumulation, and oxidative stress in corneal epithelial cells, ultimately contributing to DK (Fig. 8). These findings provide novel insights into the mechanisms underlying DK and suggest that targeting the DJ-1/PTEN/antioxidant protein signaling pathway may offer a promising therapeutic strategy.

## Supplementary Material

Supplement 1
