# Supplementary figures and images for: DJ-1 Promotes Diabetic Corneal Epithelial Wound Healing by Attenuating Hyperglycemia-Induced Oxidative Stress Through Inhibiting PTEN
Source: Invest Ophthalmol Vis Sci. 2025 Jul 8;66(9):20. doi: 10.1167/iovs.66.9.20 (PMC12248957; doi:10.1167/iovs.66.9.20)

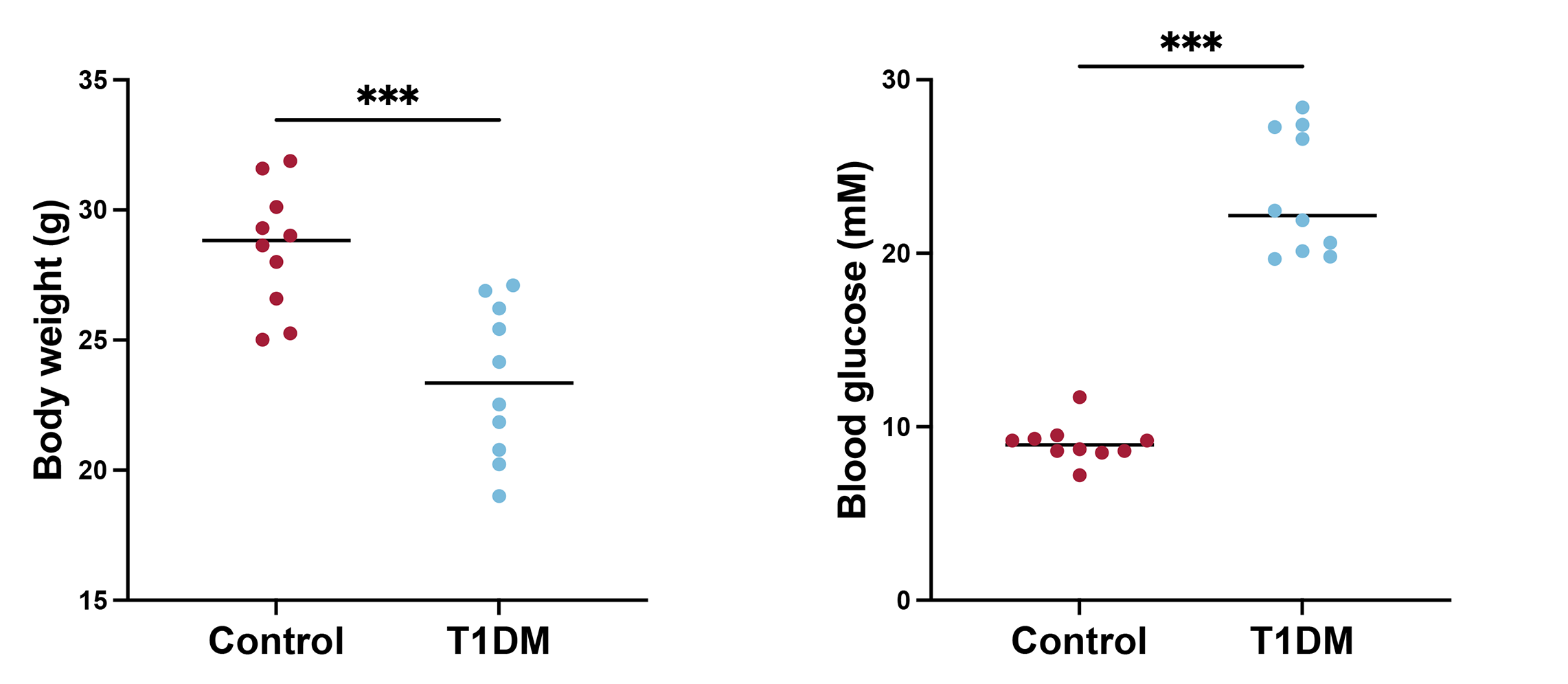

Supplement: Supplement 1 [file iovs-66-9-20_s001.tif]
